# Factors Associated with Cervical Cancer Screening among Married Female Immigrants with Korean Husbands in South Korea

**DOI:** 10.3390/ijerph15112528

**Published:** 2018-11-12

**Authors:** Ha Kyun Chang, Sang-Soo Seo, Jun-Pyo Myong, Jung-Wan Koo, Jinhee Jeong

**Affiliations:** 1 Center for Uterine Cancer, Research Institute and Hospital, National Cancer Center, 323, Ilsan-ro, Ilsandong-gu, Goyang-si, Gyeonggi-do 10408, Korea; hkchang@ncc.re.kr (H.K.C.); ssseomd@ncc.re.kr (S.-S.S.); 2 Department of Occupational & Environmental Medicine, Seoul St. Mary’s Hospital, College of Medicine, The Catholic University of Korea, 222, Banpo-daero, Seocho-gu, Seoul 06591, Korea; jwkoo@catholic.ac.kr; 3 Department of medical benefit, National Health Insurance Company, 32, Geongang-ro, Wonju-si, Gangwon-do 26464, Korea; jin87love@naver.com

**Keywords:** immigrants, cervical cancer, screening, healthcare disparities

## Abstract

*Background.* The purpose of this study was to identify factors associated with the national cervical cancer screening behaviors of married female immigrants living in South Korea. *Methods.* The present study dataset was collected by the National Health Insurance Services in 2014–2015. A final study population of 15,935 was considered eligible for inclusion in this study if they met the criteria for participation in the national cervical cancer screening program in 2014–2015. *Results.* Of the 15,935 subjects, 7837 (49%) participated in cervical cancer screening. Based on the results of the logistic regression analysis of the association between cervical cancer screening behaviors and related factors, the odds ratio (OR) for participation in cervical cancer screening among individuals older than 50 years was the highest (OR: 2.13; 95% confidence interval (CI): 1.82–2.51), and the OR increased as their duration of stay in South Korea decreased. The OR of Chinese women for cervical cancer screening participation was higher than that of non-Chinese women (OR: 1.83; 95% CI: 1.69–1.99). The OR value was 29.4 (95% CI: 25.9–33.3) among those who participated in the general health screening compared with those who did not participate. *Conclusions.* To improve awareness about cervical cancer screening and reduce disparities in access to healthcare, appropriate programs should be developed to promote cervical cancer screening participation to socially vulnerable classes. Continuous social attention is needed to address these issues and encourage participation in general health screening to improve the rate of cervical cancer screening.

## 1. Introduction

The number of married female immigrants with Korean husbands living in South Korea has been increasing and is expected to increase even further because of the specific nature of the South Korean population structure [1]. National attention and efforts are needed to help married immigrant women who are struggling to adjust to South Korean society. Additionally, married female immigrants tend to be highly dependent on their family because of their immigrant status, and this may interfere with health-related decisions, including the use of medical services. Married female immigrants often migrate from underdeveloped countries and have poor health [2]. Moreover, the income levels of women in multicultural families are lower than those of women from the host country, and studies have indicated that women who migrate tend to be among the most vulnerable population groups with respect to healthcare [3,4]. Specifically, women who do not speak the host language and are unemployed are less likely to benefit from the healthcare system of the host country [5].

Approximately 528,000 women worldwide are diagnosed with cervical cancer annually, and approximately 266,000 individuals died of this disease in 2012 [6]. In South Korea, more than 3584 new cervical cancer cases (age-standardized incidence, 9.5 per 100,000 persons) were diagnosed in 2012, accounting for 3.2% of all new female cancer cases [7]. Despite trends reflective of a decreasing incidence and mortality rate from cervical cancer, this disease remains a major public health problem. Unlike other forms of cancer, cervical cancer is associated with long-term carcinogenesis [8]. Therefore, the fact that the incidence of new cases of cervical cancer is decreasing does not necessarily mean that the future risk of cancer is lower.

Currently, there are two population-based cervical cancer screening programs in South Korea [9]. One is the National Cancer Screening Program (NCSP), whose target population includes medical aid program (MAP) recipients and National Health Insurance Service (NHIS) beneficiaries in the lower 50% income stratum. The other is the NHIS Cancer Screening Program (NHISCSP), in which the target population includes those in the upper 50% income stratum [9,10]. Together, these programs provide complimentary biennial cervical cancer screening to all South Korean women over the age of 30 years (since 2016, women aged 20–29 have been included in the cervical cancer screening program) by the Papanicolaou (Pap) smear test. It has been established that cervical cancer screening programs enable the early detection of cervical cancer, contributing to decreases in both the incidence of and mortality due to this disease [11]. Despite the government’s cervical cancer screening program, participation in cervical cancer screening is much lower in South Korea than it is in Western countries [12,13]. To increase general participation in cervical cancer screening, it is necessary to invest in increasing the participation rate. 

In Norway, immigrants showed a higher non-attendance rate for a cervical screening program (OR: 1.72 95% CI: 1.71–1.73) than did native Norwegian females [14]. Furthermore, several international studies showed that immigrants have lower participation rates in preventive screening programs for cervical cancer [15,16]. Most women immigrating to South Korea to marry South Korean men come from low- to middle income countries, making their health status relatively vulnerable compared to natives of South Korea; Japanese immigrants are one exception [17]. These female immigrants might be vulnerable to several diseases. Thus, it is important to identify the potential barriers to participation among vulnerable populations, especially female immigrants. However, few studies have evaluated the individual and environmental factors that predict participation in cervical cancer screening among married female immigrants with Korean husbands in South Korea.

The purpose of this study was to identify factors associated with the national cervical cancer screening behaviors of married female immigrants with Korean husbands living in South Korea and to establish an appropriate strategy to improve this group’s participation in cervical cancer screening.

## 2. Materials and Methods

### 2.1. Study Population

The dataset used in the present study was obtained from the National Health Insurance Services (NHIS). NHIS is the only insurance provider in South Korea. NHIS insures 51,757,146 people, including 17,379,951 of participants in national health screening, 48,309,955 people used health care in 2014 [18]. NHIS collects whole data from other departments of the government for essential information such as visa status, level of income, family structure, diseases status etc. In the present study, the authors requested data on married immigrants from the NHIS, and the NHIS provided screening, medical claim data, and basic characteristics including residence. Therefore, all of the present data were derived from the NHIS internal database registry. An F-6 type visa is issued for a marriage migrant who is to have a spouse with South Korean Nationality. To evaluate immigration status, South Korean husbands are able to qualify simultaneously by F-6 visa. A member of the study team works for the NHIS and after acquiring Institutional Review Board (IRB) approval, she accessed the NHIS database in order to estimate factors associated with participating in a health examination among female F-6 visa holders in South Korea.

All South Korean women aged 30 years or over are instructed to be screened for cervical cancer via the NHISCSP or the NCSP biennially. Participation in the NHISCSP or the NCSP tests was ascertained based on cervical cancer screening results presented to the NHIS. The NHIS has gathered data in a standardized form to check screening activities in both programs. In total, 49,786 cases met the inclusion criterion of female immigrants that married South Korean husbands in 2014–2015. The eligibility of potential participants was verified based on data regarding the NHIS marriage immigrant visa (F-6). Exclusion criteria were as follows: ineligibility for cervical cancer screening according to the National Cancer Screening Schedules (*n* = 29,838), invalid immigration date (*n* = 621), missing information about participation in cervical cancer screening (*n* = 2213), residence (*n* = 1038), socioeconomic status grade (*n* = 118), or nationality (*n* = 23). The final study population consisted of 15,935 eligible individuals.

### 2.2. Definition of Variables

For purposes of this research, participants in cervical cancer screening in 2014–2015 were individuals who were both eligible for such participation according to the National Cervical Cancer Screening Schedules for each year and who actually participated. 

Age was classified as follows: ≤39 years, 40–49 years, and ≥50 years. Data on nationality (China and other countries) are presented in Supplementary Table S1, and residence is classified as follows: capital city (Seoul), metropolitan city, and rural area. Data on socioeconomic status were obtained from the NHIS (1–20 grades) and were re-grouped into quartiles: Q1 (the lowest: 1–5) through Q4 (the highest: 16–20). The duration of participants’ stay in South Korea was defined as the difference between the date of their cervical cancer screening (for non-participants, the end of each year [31 December 2014 or 31 December 2015]) and the date of their arrival in South Korea and was grouped as follows: <5, 5–9, 10–15, or ≥15 years. Data on occupation were crosschecked with NHIS information on employment.

Comorbidities were defined as one or more known diseases (e.g., dementia, connective tissue disease, ulcer, myocardial infarction, congestive heart failure, chronic pulmonary disease, peripheral vascular disease, cerebrovascular disease, diabetes mellitus, and mild liver disease) [19] according to the Charlson’s Comorbidity Index (CCI), which is used to categorize the comorbidities of patients based on the International Classification of Diseases (ICD) diagnosis codes. The diseases were derived from the relevant subset of data from the disease content registry in the NHIS database (T40 table). Details on the NHIS database are described elsewhere [20].

### 2.3. Statistical Analysis

All statistical analyses were performed using SAS software 9.4 version (SAS Institute Inc., Cary, NC, USA). After the distribution of variables was standardized, continuous variables were subjected to *t*-tests and categorical variables were subjected to chi-square tests. Multiple logistic regression analyses were performed after adjusting for age, duration of stay, nationality, residence, economic status, eligibility for general health screening, occupation, and comorbidity. To account for the effect of general health screening, participation in the general health screening was stratified, and multiple logistic regression was performed.

### 2.4. Ethics Statement

Our study was approved by the IRB of the Seoul St. Mary’s Hospital, College of Medicine, the Catholic University of Korea (KC17ZES10589).

## 3. Results

### 3.1. General Characteristics of Subjects

The general characteristics of the study subjects are presented in Table 1. In total, 15,935 individuals were eligible for participation in this study: 7837 (49%) women participated in cervical cancer screening, and 8098 (51%) women did not participate. The mean age of the participants in cervical cancer screening was 43.5 ± 9.5 years, and the mean age of the non-participant group was 39.8 ± 9.0 years (*p* < 0.001). Older married female immigrants were more likely to participate in a cervical cancer screening than were younger female immigrants. Those who were from China, eligible for general health screening, employed (with an occupation) and with a comorbidity were more likely to participate in cervical cancer screening.

### 3.2. Logistic Regression Analysis of Factors Affecting Cervical Cancer Screening Behavior

Table 2 presents the logistic regression results for factors affecting cervical cancer screening behavior. Multiple logistic regression was performed after adjusting for age, duration of stay, nationality, residence, economic status, participation in the general health examination, occupation, and comorbidity. The logistic regression analysis of the association between cervical cancer screening behaviors and related factors indicated that the odds ratio (OR) for those older than 50 years was the highest (OR: 2.13; 95% CI: 1.82–2.51). The OR increased as the length of time in South Korea decreased. 

In terms of nationality, the OR for participation in the cervical screening program among Chinese females who underwent general health examinations was higher than that among females of other nationalities (OR: 1.83; 95% CI: 1.69–1.99). Employed women had significantly higher ORs than did unemployed women (OR: 1.20; 95% CI: 1.10–1.31). The OR value was 29.4 (95% CI: 25.9–33.3) for those who participated in the general health screening compared with those who did not. Additionally, women with a comorbidity had higher ORs for participation in cervical cancer screening than those with no diseases (OR: 1.28; 95% CI: 1.18–1.38). The results of the study showed that age, duration of stay, nationality, residence, economic status, occupation, general health screening participation, and presence/absence of a disease were significantly associated with cervical cancer screening behaviors.

### 3.3. Association Between Participation in Cervical Cancer Screening and Related Factors Stratified by Eligibility for Participation in General Health Screening

More of those who did (4675/5662 women, 82.6%) than did not (442/3261 women, 13.6%) participate in the general health screening also participated in the cervical cancer screening (Table 1). The factors associated with participation in cervical cancer screening stratified by eligibility for participation in the general health screening are presented in Table 3.

Of those who underwent the general health screening, the OR for participating in cervical cancer screening among those older than 50 years was 3.56 (95% CI: 2.81–4.51) compared with those younger than 40 years. Among those individuals who did not participate or were not eligible for participation in the general health screening, employed women who had participated in the general health screening had significantly lower ORs than did unemployed females who did not participate (OR: 0.69, 95% CI: 0.58–0.81).

## 4. Discussion

To our knowledge, this is the first study to identify factors associated with the national cervical cancer screening behaviors of married female immigrants with Korean husbands living in South Korea. Among the 15,935 subjects, 7837 (49%) participated in cervical cancer screening, which was lower than the screening rate of 54% of women reported by the statistical yearbook of health screening in 2015 [21]. According to the logistic regression analysis of the association between cervical cancer screening behaviors and related factors, age, duration of stay, nationality, residential area, economic status, occupation, general health screening participation, and presence/absence of a comorbidity were associated with screening for cervical cancer.

The general health screening aims to improve public health and reduce medical expenses by the early detection and prevention of non-communicable diseases, including metabolic syndrome and its related basic diseases. According to the current study, 83% of the women who participated in the general health screening underwent cervical cancer screening, and only 14% of people who did not participate in general health screening received a cervical cancer screening. A high rate of participation in general health screening is likely to have a significant impact on cancer screening because people who generally undergo general health screening are more likely, than those who generally do not, to be interested in their health, and the former try to obtain regular checkups [22]. Therefore, women who participate in general health screenings are more willing to attend a cervical cancer screening. It is thought that women who attend the general health screening and simultaneously participate in cervical cancer screening can also save time. Hence, participation in general health screening can also be considered an important contributor to participation in cervical cancer screening.

Employed female immigrants had a higher participation rate in cervical cancer screenings than did unemployed female immigrants. This is because employers are mandated to sponsor health checkups to protect their employees’ health; otherwise, the Ministry of Employment and Labor imposes penalties in accordance with Article 72 of the Industrial Safety and Health Act [23]. Therefore, employers encourage participation in cervical cancer screening. This is in line with previous research showing that women in the workplace are more likely to be screened than women who do not work [14,24,25]. Patients who visited a clinic for a medical checkup were likely to be recommended for a cervical cancer screening.

The effects of employment may be different after stratification using a general health screening. Employed patients who participated in a general health checkup were not likely to participate in cervical cancer screenings. In South Korea, mobile mass screenings for general health for working populations who work for a company are allowed. However, cervical cancer screening chairs are not installed in screening vehicles, so workers that participate in a health checkup in the workplace may lose the opportunity to participate in cervical cancer screenings. Immigrant female workers who do not participate in or who are not eligible for a general health checkup can visit a clinic for a cervical cancer screening. Therefore, working people who are eligible for both a general health checkup and cervical cancer screening should visit a non-mobile clinic that provides both a general checkup and cervical cancer screening.

Many previous studies on unequal access to healthcare as a function of socioeconomic status [15,26] have emphasized the vulnerability of low-income women, irrespective of immigration status. According to Sung et al., members of socioeconomically vulnerable groups have reported that they lacked the time and money to participate in health screening [22]. Consistent with previous studies, the current research found that the low-income group reported relatively weak intentions to undergo cervical cancer screening due to a lack of awareness about the necessity of health checkups; this group also devoted scant attention to their own healthcare due to their socioeconomic situation. Therefore, to enhance the awareness of the importance of healthcare and to improve the rate of participation in health screenings, we must create and implement policies that promote health checkups for socioeconomically vulnerable people; this will require the development of a social consensus about and continuous attention to the importance of providing healthcare to all people.

A previous study found that women from China were the most likely to participate in regular health screenings [27,28]. In the present study, Chinese women had the highest ORs for participation in cervical cancer screenings. China is one of the countries nearest to South Korea, and the majority of immigrants to South Korea come from China [29]. A supportive network among immigrants is formed in South Korea, and these networks enable immigrants to exchange information about participating in screenings [30]. Additionally, Chinese women have high levels of family support, which has a positive effect on participation in health screening behaviors. In view of the significant differences in participation in cervical cancer screenings by country of origin, programs that encourage screening should consider the social and cultural characteristics of each country.

The duration of stay in the host country may be a significant predictor of whether an immigrant undergoes cervical cancer screening. Several studies have reported that a longer duration since immigration is associated with an increased likelihood of participation in screenings [16,24,31]. In contrast, the results of our study indicate that a long duration of stay is a barrier to receiving cervical cancer screenings. In addition, it is possible that women who have spent more time in South Korea may be less likely to participate in cervical cancer screening. Additional analyses of the duration of stay and nationality (data not shown) indicate that immigrants from China had immigrated later than those from other countries (the proportion of Chinese immigrants in South Korea for less than 180 months (58.2%) is higher than the proportion in South Korea for more than 180 months (10.6%)). Therefore, it would be prudent to provide immigrants with culturally sensitive and specific information to overcome any barriers to participation in screening programs.

In this study, the rate of cervical cancer screening was significantly higher among those with comorbidities than those without other conditions. This may be because women who have already been diagnosed with a disease may receive information about healthcare from their hospital and may recognize the need for screening. Women with health conditions may also be more interested in health and more likely to receive a recommendation from medical staff for a health examination.

This study has several limitations. First, because the customized database of the NHIS was used as the source of information on comorbidities as well as a way to determine eligibility to participate in this study, we were unable to examine all potential variables of interest. For example, data on several potentially influential variables, such as educational level, social support, and life satisfaction were not available. However, we attempted to gather data on these variables (e.g., duration of residence and nationality) from secondary sources. Second, because our selection criteria included all women with a marriage visa at the time of cervical cancer screening, women who had changed their visa, acquired South Korean nationality or were married to non-South Korean men were excluded. Third, certain clinical and demographic variables (e.g., education, health insurance type, current marital status, disability, occupation, region, and tobacco use) that may influence participation in cervical cancer screenings were not addressed. Fourth, the prevalence ratio for participation in cervical cancer screenings between married female immigrants and other women was not assessed due to restricted access to the database registry. Despite these limitations, this study is the first to analyze the factors affecting participation in cervical cancer screening among married female immigrants with Korean husbands living in South Korea.

## 5. Conclusions

To improve awareness about the importance of cervical cancer screening and to overcome disparities in access to healthcare, we need to develop and implement appropriate programs to promote cervical cancer screening among socially vulnerable classes, especially for younger, unemployed female immigrants who have lived in South Korea for a relatively long period, have no comorbidities, and have no interest in healthcare. Continuous social attention to this issue is needed, and participation in general health screening should be encouraged to improve the rate of cervical cancer screening. Further research on health screening will be necessary to improve the health of those who have become members of our society.

## Figures and Tables

**Table 1:** Descriptive statistics by cervical cancer screening participation among married immigrant females with South Korean husband 2014–2015.

Variables	Cervical Cancer Participation			*p*-Value ^a^
	Yes (*n* = 7837)	No (*n* = 8098)	Total (*n* = 15935)	
Age (years) ^b^	43.5 (9.5)	39.8 (9.0)	41.6 (9.5)	<0.0001
≤39	3001 (38.3%)	4840 (61.7%)	7841 (49.2%)	<0.0001
40–49	2605 (56.8%)	1978 (43.2%)	4583 (28.8%)	
≥50	2231 (66.4%)	1280 (36.5%)	3511 (22.0%)	
Duration of stay (months)	77.4 (41.0)	81.8 (43.4)	79.6 (42.3)	<0.0001
<5 years	2876 (52.4%)	2610 (47.6%)	5486 (34.4%)	<0.0001
5–9 years	3934 (47.7%)	4305 (52.3%)	8239 (51.7%)	
10–14 years	866 (48.4%)	925 (51.6%)	1791 (11.2%)	
≥15 years	161 (38.4%)	258 (61.6%)	419 (2.6%)	
Nationality				
China	5314 (58.6%)	3753 (41.4%)	9067 (56.9%)	<0.0001
Other^c^	2523 (36.7%)	4345 (63.3%)	6868 (43.1%)	
Residence				
Capital city	1902 (53.8%)	1636 (46.2%)	3538 (22.2%)	<0.0001
Urban city	1526 (51.9%)	1412 (48.1%)	2938 (18.4%)	
Rural city	4409 (46.6%)	5050 (53.4%)	9459 (59.4%)	
Economic status ^d^				
Q1 (lowest)	2067 (50.4%)	2033 (49.6%)	4100 (25.7%)	<0.0001
Q2	2974 (50.4%)	2930 (49.6%)	5904 (37.1%)	
Q3	1992 (48.8%)	2086 (51.2%)	4078 (25.6%)	
Q4 (highest)	804 (43.4%)	1049 (56.6%)	1853 (11.6%)	
Occupation				
No	5342 (46.5%)	6154 (53.5%)	11496 (72.1%)	<0.0001
Yes	2495 (56.2%)	1944 (43.8%)	4439 (27.9%)	
General health screening				
Participated	4675 (82.6%)	987 (17.4%)	5662 (20.5%)	<0.0001
Not participated	442 (13.6%)	2819 (86.4%)	3261 (35.5%)	
Not eligible subject	2720 (38.8%)	4292 (61.2%)	7012 (44.0%)	
Comorbidity ^e^				
No	4455 (44.3%)	5601 (55.7%)	10056 (63.1%)	<0.0001
Yes	3382 (57.5%)	2497 (42.5%)	5879 (36.9%)	

^a^ The *t*-test and chi-squared were used to assess the significance of subjects’ differences in continuous and categorical variables, respectively. ^b^ Age at eligible subjects for cervical cancer screening. ^c^ Vietnam, Philippines, Japan, Mongolia and Thailand are included in “other.” ^d^ Q1 (lowest): 1–5th income class; Q2: 6–10th income class; Q3: 11–15th income class; Q4 (highest): 16–20th income class derived from NHIS equation with subject’s asset profiles. ^e^ Charlson Comorbidity Index weigh 1 (Dementia, Connective tissue disease, Ulcer disease, Myocardial infarction, Congestive heart failure, Chronic pulmonary disease, Peripheral vascular disease, Cerebrovascular disease, Diabetes mellitus, Mild liver disease).

**Table 2:** The association between cervical cancer screening participation and related factors.

Variables	Simple Logistic Regression OR (95% CI)	Multiple Logistic Regression ^a^ OR (95% CI)
Age (years) ^b^		
≤39	1 (ref)	1 (ref)
40–49	2.12 (1.97–2.29)	2.05 (1.77–2.36)
≥50	2.81 (2.59–3.05)	2.13 (1.82–2.51)
Duration of stay (years)		
<5	1.77 (1.44–2.17)	1.50 (1.14–1.99)
5–9	1.46 (1.20–1.79)	1.19 (0.90–1.56)
10–14	1.50 (1.21–1.87)	1.11 (0.83–1.48)
≥15	1 (ref)	1 (ref)
Nationality		
China	2.44 (2.29–2.60)	1.83 (1.69–1.99)
Other ^c^	1 (ref)	1 (ref)
Residence		
Capital city	1 (ref)	1 (ref)
Urban city	0.93 (0.84–1.03)	1.14 (1.01–1.29)
Rural city	0.75 (0.70–0.81)	0.86 (0.78–0.94)
Economic status ^d^		
Q1 (lowest)	1 (ref)	1 (ref)
Q2	1.00 (0.92–1.08)	1.12 (1.01–1.24)
Q3	0.94 (0.86–1.02)	1.25 (1.12–1.40)
Q4 (highest)	0.75 (0.68–0.84)	1.11 (0.97–1.28)
Occupation		
No	1 (ref)	1 (ref)
Yes	1.48 (1.38–1.59)	1.20 (1.10–1.31)
General health screening		
Participated	30.2 (26.8–34.1)	29.4 (25.9–33.3)
Not participated	1 (ref)	1 (ref)
Not eligible subject	7.47 (6.88–8.13)	8.19 (6.97–9.62)
Comorbidity ^e^		
No	1 (ref)	1 (ref)
Yes	1.70 (1.60–1.82)	1.23 (1.18–1.38)

OR: odds ratio, CI: confidence interval. ^a^ Adjusted for age, duration of stay, nationality, residence, economic status, occupation, general health examination and comorbidity were performed for multiple logistic regression. ^b^ Age at eligible subjects for cervical cancer screening. ^c^ Vietnam, Philippines, Japan, Mongolia and Thailand are included in “other.” ^d^ Q1 (lowest): 1–5th income class; Q2: 6–10th income class; Q3: 11–15th income class; Q4 (highest): 16–20th income class derived from NHIS equation with subject’s asset profiles. ^e^ Charlson Comorbidity Index weigh 1 (Dementia, Connective tissue disease, Ulcer disease, Myocardial infarction, Congestive heart failure, Chronic pulmonary disease, Peripheral vascular disease, Cerebrovascular disease, Diabetes mellitus, Mild liver disease).

**Table 3:** The association between cervical cancer screening participation and related factors stratified by general health screening.

Variables	General Health Screening	
	Participated OR (95% CI) ^a^	Not Participated + Not Eligible OR (95% CI) ^a^
Age (years) ^b^		
≤39	1 (ref)	1 (ref)
40–49	3.37 (2.73–4.16)	1.30 (1.00–1.68)
≥50	3.56 (2.81–4.51)	0.92 (0.62–1.37)
Duration of stay (years)		
<5	1.09 (0.71–1.63)	2.47 (1.56–4.11)
5–9	1.09 (0.72–1.62)	1.76 (1.12–2.93)
10–14	1.12 (0.72–1.72)	1.50 (0.93–2.54)
≥15	1 (ref)	1 (ref)
Nationality		
China	1.88 (1.57–2.24)	1.73 (1.57–1.90)
Other ^c^	1 (ref)	1 (ref)
Residence		
Capital city	1 (ref)	1 (ref)
Urban city	0.94 (0.74–1.20)	1.29 (1.12–1.48)
Rural city	0.70 (0.58–0.84)	0.99 (0.88–1.11)
Economic status ^d^		
Q1 (lowest)	1 (ref)	1 (ref)
Q2	1.13 (0.96–1.34)	1.23 (1.09–1.39)
Q3	1.25 (1.01–1.55)	1.38 (1.21–1.57)
Q4 (highest)	1.19 (0.88–1.64)	1.24 (1.06–1.45)
Occupation		
No	1 (ref)	1 (ref)
Yes	1.84 (1.67–2.04)	0.69 (0.58–0.81)
Comorbidity ^e^		
No	1 (ref)	1 (ref)
Yes	1.36(1.24–1.50)	1.06 (0.91–1.23)

OR: odds ratio, CI: confidence interval. ^a^ Adjusted for age, duration of stay, nationality, residence, economic status, occupation and comorbidity were performed for multiple logistic regression. ^b^ Age at eligible subjects for cervical cancer screening. ^c^ Vietnam, Philippines, Japan, Mongolia and Thailand are included in “other”. ^d^ Q1 (lowest): 1–5th income class; Q2: 6–10th income class; Q3: 11–15th income class; Q4 (highest): 16–20th income class derived from NHIS equation with subject’s asset profiles. ^e^ Charlson Comorbidity Index weigh 1 (Dementia, Connective tissue disease, Ulcer disease, Myocardial infarction, Congestive heart failure, Chronic pulmonary disease, Peripheral vascular disease, Cerebrovascular disease, Diabetes mellitus, Mild liver disease).

## Supplementary Materials

The following are available online at http://www.mdpi.com/1660-4601/15/11/2528/s1, Table S1: Nationality of eligible population.
