# Supplementary material for: Factors Associated with Cervical Cancer Screening among Married Female Immigrants with Korean Husbands in South Korea
Source: Int J Environ Res Public Health. 2018 Nov 12;15(11):2528. doi: 10.3390/ijerph15112528 (PMC6266390; doi:10.3390/ijerph15112528)
Supplement: Supplementary file 1 [file ijerph-15-02528-s001.pdf]

International Journal of *Environmental Research and Public Health*

MDPI

Table S1. Nationality of eligible population.

Nationality	N (%)
**China**	9067 (56.90)
**Vietnam**	2773 (17.40)
**Philippines**	1098 (6.89)
**Japan**	1031 (6.47)
**Mongolia**	359 (2.25)
**Thailand**	353 (2.21)
**Cambodia**	278 (1.74)
**Uzbekistan**	198 (1.24)
**Russian Federation**	190 (1.19)
**United States**	98 (0.61)
**Taiwan, China**	66 (0.41)
**Nepal**	59 (0.37)
**Indonesia**	56 (0.35)
**Kyrgyz Republic**	41 (0.26)
**Kazakhstan**	36 (0.23)
**Canada**	26 (0.16)
**Hong Kong SAR, China**	21 (0.13)
**Pakistan**	19 (0.12)
**Lao PDR**	17 (0.11)
**Malaysia**	13 (0.08)
**Bangladesh**	12 (0.07)
**United Kingdom**	9 (0.06)
**Peru**	8 (0.05)
**Myanmar**	8 (0.05)
**Sri Lanka**	6 (0.04)
**Turkey**	6 (0.04)
**Australia**	6 (0.04)
**Mexico**	5 (0.03)
**Ukraine**	5 (0.03)
**Belarus**	4 (0.03)
**Austria**	4 (0.03)
**France**	4 (0.03)
**Guatemala**	3 (0.02)
**Morocco**	3 (0.02)
**Moldova**	3 (0.02)
**Singapore**	3 (0.02)
**India**	3 (0.02)
**South Africa**	2 (0.01)
**Netherlands**	2 (0.01)
**New Zealand**	2 (0.01)
**Romania**	2 (0.01)
**Bulgaria**	2 (0.01)
**Brazil**	2 (0.01)
**Argentina**	2 (0.01)
**El Salvador**	2 (0.01)
**Ethiopia**	2 (0.01)
**Italy**	2 (0.01)
**Congo, Rep.**	2 (0.01)

**Ghana**	1 (0.006)
**Guyana**	1 (0.006)
**Greece**	1 (0.006)
**Norway**	1 (0.006)
**Germany**	1 (0.006)
**Benin**	1 (0.006)
**Bolivia**	1 (0.006)
**Burundi**	1 (0.006)
**Swaziland**	1 (0.006)
**Spain**	1 (0.006)
**Ireland**	1 (0.006)
**Ecuador**	1 (0.006)
**Honduras**	1 (0.006)
**Uruguay**	1 (0.006)
**Iran, Islamic Rep.**	1 (0.006)
**Djibouti**	1 (0.006)
**Chad**	1 (0.006)
**Colombia**	1 (0.006)
**Paraguay**	1 (0.006)
**Poland**	1 (0.006)
**Finland**	1 (0.006)
**Hungary**	1 (0.006)
